# Using Convolutional Neural Networks with Multiple Thermal Sensors for Unobtrusive Pose Recognition

**DOI:** 10.3390/s20236932

**Published:** 2020-12-04

**Authors:** Matthew Burns, Federico Cruciani, Philip Morrow, Chris Nugent, Sally McClean

**Affiliations:** School of Computing, Ulster University, Belfast BT37 0QB, UK; f.cruciani@ulster.ac.uk (F.C.); pj.morrow@ulster.ac.uk (P.M.); cd.nugent@ulster.ac.uk (C.N.); si.mcclean@ulster.ac.uk (S.M.)

**Keywords:** CNN, thermal, smart environment, pose recognition, sensors, deep learning

## Abstract

The desire to remain living in one’s own home rather than a care home by those in need of 24/7 care is one that requires a level of understanding for the actions of an environment’s inhabitants. This can potentially be accomplished with the ability to recognise Activities of Daily Living (ADLs); however, this research focuses first on producing an unobtrusive solution for pose recognition where the preservation of privacy is a primary aim. With an accurate manner of predicting an inhabitant’s poses, their interactions with objects within the environment and, therefore, the activities they are performing, can begin to be understood. This research implements a Convolutional Neural Network (CNN), which has been designed with an original architecture derived from the popular AlexNet, to predict poses from thermal imagery that have been captured using thermopile infrared sensors (TISs). Five TISs have been deployed within the smart kitchen in Ulster University where each provides input to a corresponding trained CNN. The approach is evaluated using an original dataset and an F1-score of 0.9920 was achieved with all five TISs. The limitations of utilising a ceiling-based TIS are investigated and each possible permutation of corner-based TISs is evaluated to satisfy a trade-off between the number of TISs, the total sensor cost and the performances. These tests are also promising as F1-scores of 0.9266, 0.9149 and 0.8468 were achieved with the isolated use of four, three, and two corner TISs, respectively.

## 1. Introduction

There is a growing need for environments that not only facilitate 24/7 care but also fulfil the desire for one’s privacy at home. An environment that can provide such necessities is known as a smart environment and has been defined as being able to not only acquire but also apply the knowledge gained from the environment and those in it to improve the experience within the environment [1]. Effectively providing automated care within an environment while providing sufficient privacy can be a challenging and potentially contradictory objective. The need for this type of care environment arises from the frequently highlighted fact that the world’s population will increase from 7.7 to 8.5 billion by 2030 and that by 2050 the number of people aged over 65 will outnumber those aged from 15–24, with projections showing that 16% of the world’s population will be aged 65 or older [2]. It can, therefore, be anticipated that the number of people requiring care (either in their own home or a secure and safe environment) will increase and so the need to provide “aging in place” will become more significant. Findings have shown that to elderly people, “aging in place” means that they would be able to continue to live with a degree of independence and autonomy [3]. Facilitating feelings of familiarity and security have been shown to be important aspects of providing “aging in place” and so allowing someone to remain living at home rather than a care home is vital. This has been reinforced by the fact that those who have moved into institutionalised life have often lost motivation for their own self autonomy and experienced feelings of isolation and depression [4].

Delivering the capability to provide 24/7 monitoring of those in need of care would significantly benefit the healthcare professionals who have responsibility for such care. Expecting healthcare professionals to be able to provide 24/7 care is neither realistic nor cost effective. Installing sensors within the home is one way to allow for automated monitoring. Video cameras could potentially be used, however, this would contradict the previously stated need for privacy as concerns for the lack of privacy provided by video cameras have previously been expressed [5]. Thermal sensors could provide a balance between descriptive data and privacy as images of the environment and its inhabitants would be able to be captured while omitting any of their discernible features.

For suitable monitoring, it would be necessary to use sensors integrated throughout the home to recognise and understand inhabitants’ actions in the context of daily life within the home. Such an action is called an Activity of Daily Living (ADL) and covers all activities performed in full autonomy in day to day life. Recognising the ADLs of a home’s inhabitants can, e.g., allow for accurate detection of abnormal behaviour during the completion of activities, resulting in the detection of cognitive decline [6]. Identifying such decline and providing warnings can be highly beneficial to caregivers. Prior to detecting ADLs, it is important to understand how they are constructed as each ADL consists of subactivities. An example of this can be highlighted by the ADL “making a tea/coffee,” where the ADL itself can be made up of tasks such as “using the fridge, “opening the coffee cupboard” and “using the kettle.” There are significant differences between each of these subactivities in terms of the inhabitant’s body shape or “pose” when performing the subactivities. In this case, thermal-based sensors could be used to classify the poses as they can effectively detect sources of heat, without identifying discernible characteristics of the inhabitant, and thus provide a detailed and unobtrusive description of the inhabitant’s pose. Accurately recognising the poses being performed can, therefore, be extremely helpful in understanding the ADL being performed and potentially, the quality of its execution in relation to assessments such as the Katz index [7].

There is potential that the accuracy and efficiency of providing “aging in place” can suffer due to the common need for privacy measures, however, the unobtrusive and low-resolution nature of the thermal sensors used in this work are a viable solution. Attempting automated and constant care using sensors can, however, involve practical limitations of integrating sensors within the environment. The challenge, therefore, is to find the correct balance between accuracy, privacy and practicality in the approach to monitoring a smart environment.

The primary contributions of the research described in this paper are:

1. Investigation of the capabilities of utilising five thermal sensors to provide multiple perspectives of a smart environment in conjunction with a Convolutional Neural Network to provide an unobtrusive and noninvasive approach to recognising full body poses/actions.
2. Practical limitations of using a ceiling sensor are considered and the use of only the sensors providing lateral views is tested. The various permutations and combinations of the sensors are investigated to determine the trade-off between performance, cost and the number of required sensors.

The remainder of the paper is structured as follows: Section 2 presents background and related work; Section 3 discusses the proposed methodology for pose recognition using deep learning and describes the experiments conducted to find the best performing sensor permutation; Section 4 presents an analysis of the results obtained from the experiments; finally, Section 5 presents discussion and conclusions along with details of limitations and future work.

## 2. Background

To understand the behaviour of an environment’s inhabitant, it is important to detect and recognise the ADLs that are performed within the environment. Sensors can be used to collect data from known performances of ADLs to train machine learning algorithms to recognise the same ADLs from future unseen data. Some sensors that can be used to collect this descriptive data are wearable and image capture devices. Wearable devices can be placed on a person’s body to collect data as they perform actions or ADLs. The data captured for each action acts almost as a fingerprint in its uniqueness for classification tasks in which an inhabitant’s actions must be described to a machine learning algorithm.

Body worn accelerometers can be used to collect data that is descriptive of the wearer’s actions. The pose or activity being performed can be represented numerically in the form of features extracted from the sensor data. The study in [8] used body worn accelerometers to capture physiological data from the performances of activities such as walking, running and jumping. The signal retrieved by the sensors was first segmented to determine the beginning and ending of the activity and from this segment, various features were extracted. The extracted features most useful for the classification of the activity were then selected and any remaining features were deemed redundant and subsequently discarded. The selected features were provided as the input for the random forest and C4.5 decision tree machine learning algorithms where the output was a prediction of the activity class label. Once their respective results were compared, it was found that the random forest produced the marginally better performance with a 99.90% recognition rate, whereas the C4.5 decision tree achieved 99.87%.

Wearable devices were also successfully integrated for gesture recognition in [9]. In this study, a flexible bracelet for electromyography (EMG) gesture recognition that boasted flexible solar panels to charge the battery was proposed. The device consisted of four EMG sensors, and a selection of features for EMG preprocessing were extracted for the classification problem. Of the tested features, the Discrete Wavelet Transform (DWT) achieved the highest accuracy score and so was used in the final application. The Support Vector Machine (SVM) was chosen as the classification algorithm to train and test where the data was broken down into three datasets made up of different volunteers. To determine the overall performance, fivefold cross-validation was applied. The five classes of gesture that were targeted in this study were rest position, hand open, power grasp and pronation and supination of the wrist. The experiment consisted of 3 people performing gestures while wearing the device. An average accuracy of 94% was achieved while avoiding being intrusive in nature. Issues, however, may arise from the utilisation of wearable sensors for gesture recognition solutions if the inhabitant forgets to wear the device or decides not to wear it at all due to discomfort [10]. In the case of elderly people, the residents may not accept such a system due to a lack of user friendliness and the need for specific training for using the device [10,11]. The sensor’s battery life and constant need for recharging can also limit the implementation of such a solution where there is a necessity for 24/7 monitoring within the home [12].

Image capture devices such as visible spectrum video cameras can detect a person’s full range of motion and so can be utilised to recognise the actions performed within the home. The importance of this recognition capability is indicated by the study completed in [13] as detecting abnormal behaviour can subsequently highlight potential health problems. An RGB-D video camera was positioned in the ceiling to provide a top-down view of the environment where the camera was used to collect image and depth data to locate the person as well as the 3D positions of their head and hands in each frame. Sequences of the 3D positions were analysed during the completion of various activities so that the Hidden Markov Model (HMM) could be trained to recognise activities from such head and hand position sequences. An F1-score of 0.8000 was achieved with the HMM that utilised only the sequence of head positions for its input.

An analysis on 3D posture data was conducted in [14], which included the recognition of various gestures using an RGB-D camera. A Microsoft Kinect was used to capture images of the person from which numerous joints were detected that would act as the features to aid in the classification of the posture. A K-means clustering method detected the postures and for the classification of each posture, an SVM was used. Each activity to be classified was defined as a unique sequence of known postures. In one of the tests performed on the novel dataset, Kinect Activity Recognition Dataset, the 18 classes of activities were split into two categories: gestures and actions. The gestures involved examples such as *Bend*, *High Arm Wave* and *Horizontal Arm Wave* while the actions included *Drink*, *Phone Call* and *Walk*. The test was conducted to find how accurately the image-based system could classify the gestures and actions that were based on similar postures. After the data samples of each subject were split into two-thirds training and one-third testing, the gestures were recognised with an accuracy of 93.00%.

For classification tasks, there are various machine learning algorithms to select from. Machine learning algorithms, such as SVMs and random forests, utilise a training dataset from which descriptive and unique features are extracted. It is important that the extracted features are effective at training the algorithm to accurately predict the classes within unseen data samples [15]. Nevertheless, [16] states that once the task of extracting features becomes almost as challenging as the problem for which the machine learning model is used, this method of learning may not be suitable. Alternatively, a more advanced manner of learning called deep learning can be used. Deep learning algorithms obtain knowledge from experience where simple concepts are used to build complex concepts. For example, corners and contours are simple concepts that can be combined to be defined as edges, subsequently representing the concept of a person. It is also detailed in [16] that concepts are built on top of one another, establishing multiple layers and a “deep” architecture.

A Convolutional Neural Network (CNN) is a type of deep learning neural network designed to process series and image data. With the capability of automatically selecting features from the input data, it can be useful to employ a CNN for recognition-based tasks involving images of 3D shapes. Such tasks have previously been fulfilled using CNNs to recognise 3D shapes [17]. This work was motivated by the theory that rather than using 3D shape descriptors, a 3D shape could be better recognised using a series of views of the 3D shape rendered as 2D images. The Multiview CNN (MVCNN) was designed by capturing 12 unique 2D views of the 3D shape that was to be recognised and inputting the 2D images separately into the CNN. View-based features were then extracted and inputted into a view-pooling layer. The shape descriptor was finally obtained by passing the extracted features through the final part of the network. The network primarily consisted of five convolutional layers, three fully connect layers and a softmax classification layer. After pretraining the network with ImageNet [18], all of the 2D views for each 3D shape were used to finalise the training. The work was evaluated on the Princeton ModelNet dataset [19] and compared against various other works. The MVCNN outperformed each of the state-of-the-art descriptors. The MVCNN was fine-tuned on the 2D views of the 3D shapes from the ModelNet training set. This fine-tuning bolstered the performance, obtaining 89.9% classification accuracy. This accuracy was a 12.8% increase from a state-of-the-art 3D shape descriptor, demonstrating how 3D objects within an environment can be successfully recognised using a CNN without the need of 3D shape descriptors. Using multiple 2D images representing various views of the object were instead shown to be a superior solution.

As there is potential for video cameras to have an intrusive nature [5] a different method of image capture can be facilitated to avoid this. Various approaches for the preservation of privacy using thermal imagery have been proposed, such as preserving privacy during the digitisation of the thermal image, altering the sensor noise to remove facial features and the use of exposure bracketing to preserve privacy in thermal high-dynamic range (HDR) images [20]. In the first approach, sensor values that fell within the human temperature range were detected as the image was digitised and so their pixels were zeroed. This prevented any successful recognition of a person’s face. The second approach was also conducted during the creation of the image where microbolometer voltages and gain amplification of the thermal device were altered to permit only image noise that would hide a person’s facial features. The same gesture dataset consisting of 20 examples of 10 hand gesture classes was used for both approaches to determine how well the gestures could be classified following the approach’s application. A multiclass bag-of-words SVM-based classifier was trained, and an accuracy of 97% was achieved with both approaches. In the third approach, areas within a thermal image equating to the temperature range of human skin were removed by either overexposing or underexposing the pixels, while HDR is maintained throughout the rest of the image.

There has been a concern highlighted that even if a system was not designed to report images, the images could still be accessed if the system was hacked. The study in [21] proposed an approach to detecting and identifying people while the captured data preserved their privacy. The system, called Lethe, used limited memory storage and data transmission so that full thermal images would not be compromised in the event of the system being hacked. Two thermal cameras were stacked on top of one another in a door frame to collect data as people walked through the door. Values that fell within the range of human skin temperature were used for person detection where the direction of movement was determined by comparing the current frame with the previous frame. Height was chosen as the feature to detect a person’s identity as only the pixel representing the top of a person’s head, within a single thermal camera’s Field of View (FOV), was required to determine their height. Both cameras calculated a height value, which was used alongside the person’s distance from the cameras to estimate their physical height. The experiment found that Lethe could predict the direction of a person’s movement with an overall accuracy of 99.70%. In a best case example, 3 participants were identified, as they passed though the doorway several times, with an accuracy of 99.10%. Pairs of participants, with a difference of 5 cm or greater in their height when walking, were identified with 96.00% and an accuracy of 92.90% was achieved for differences of 2.5 cm. In total, 21 values were required in the thermal camera’s memory for this approach, resulting in a memory requirement of 33 bytes. Only 0.69% of an image could, therefore, ever be stored on the 60 × 80 pixel thermal camera that was used in this study. A low data rate hardware transmitter was also used in the attempt to preserve privacy. The transmissions from the device were expected to last a minimum of 0.366 s, therefore, the data rate was only required to be 30 bytes per second. The limited memory storage alongside the low data rate ensured that full thermal images could not be taken from the device.

Thermopiles use thermal energy as their input in order to output a voltage in the range of tens of hundreds of millivolts. This voltage is directly proportional to the local temperature difference on the thermopile. The thermopiles can be used to provide a level of spatial temperature averaging [22], therefore, the imaging solution can be realised and has been done so through the development of thermopile 2D arrays [23]. The thermopile infrared sensor (TIS) devices generate temperature values of the local space that are stored within arrays whose sizes range from 8×8 to 120x84 The temperature values generated at any given instant can be used to generate a thermal image by scaling the values to the range of 0–255. In such a case, each value would represent a pixel’s grey level. This would result in the hottest aspects of the image being represented by pixels closer to the upper limit of the range (white) with the colder portions being represented by pixels closer to the lower limit (black). The thermal images produced can clearly show the heat signature of the person within a monitored space, however, no discernible characteristics of the person’s body or their face can be seen [24].

In contrast to the previously highlighted privacy preserving approaches, our work utilises the low-resolution images captured by TISs. High-resolution thermal images have proven to be advantageous while preserving privacy [20,21] where the facial features of a person were successfully hidden while maintaining a high-quality image for the rest of the scene. A high resolution was particularly practical in [21] as identifying the pixel representing the top of a person’s head allowed for the eventual estimation of their height, therefore, facilitating the identification of the person. A low-resolution image would not allow for an accurate determination of the top of the head, subsequently hindering the system’s capability to use height to accurately differentiate between people. For our work, however, only the differences in shape of the inhabitant’s heat signature between different pose classes and their position within the frame are needed from the data, therefore, the TIS provides sufficient information. The low resolution of the data benefits our approach as no image processing techniques are necessary to remove characteristics of the person’s face from the data, therefore, if a system incorporating TIS data was hacked, privacy would still be preserved. Unlike the previously detailed work that is able to maintain a high resolution for the environment within the images, the low-resolution TIS data is unable to include details of the environment. This feature is not, however, necessary for our work’s application area and, instead, our work benefits from a lack of environmental detail as images of one’s home cannot be recorded, subsequently improving the privacy preservation.

In our previous work with TISs, we proposed an approach for inferring basic activities performed within a smart kitchen, such as using the fridge and sitting at the kitchen table [25]. This was accomplished by combining knowledge of the inhabitant’s pose with the object that was “nearest” to the person at the time of the pose performance. The “nearest” object was determined using the ceiling TIS frame to calculate the Euclidean distance between the person and each kitchen object, whereby if the shortest distance was less than an empirically chosen threshold, the person was deemed “near” the object. The pose prediction and “nearest object” calculation pairing was used in conjunction with one another to infer the most likely activity being performed, e.g., when the person bent down while near the fridge, it was inferred that the fridge was being used.

Seven poses were selected for classification: left arm extended forwards, right arm extended forwards, left arm extended sideward, right arm extended sideward, bending, sitting and both arms down. Random forest, SVM and complex decision tree machine learning models were each utilised to classify the poses and a comparison was conducted where the random forest was found to be the best performing model. Both the training data and the testing data were captured using two TISs integrated within the environment. One TIS was embedded in the ceiling to provide a top-down perspective of the pose performances and one was positioned in a corner of the room to provide a lateral perspective. For each pose performance, the person’s heat signature was detected within both the ceiling TIS frame and the corner TIS frame. From the person’s heat signature in both TIS frames, 14 unique features were extracted, upon which both features vectors were combined to result in a feature vector consisting of 28 feature values. This feature vector was used as the random forest’s input where a prediction for the pose was the subsequent output. The poses were classified at a rate of 88.91% and the nearest objects were calculated with an accuracy of 81.05%. The activities were inferred with an accuracy of 91.47%, therefore, demonstrating how low-resolution thermal imagery could be effectively utilised for the prediction of poses and, resultantly, the inference of activities.

As shown by our previous work, using TIS data to recognise the poses being performed can provide an understanding of the activities an inhabitant conducts within the smart environment, while preserving privacy. It was believed, however, that the pose recognition rate could be significantly improved from what was achieved in our previous work. The work conducted in this paper, therefore, introduces two major changes to the previous pose recognition approach. The first change was that the number of TISs deployed in the smart kitchen was increased from two to five. It was hypothesised that introducing the means of capturing additional perspectives of each pose performance would reduce misclassifications that resulted from either the person not being detected by a TIS or part of their pose being occluded. How CNNs could be used with the TIS data in place of the random forest machine learning algorithm was the second change. Where a single random forest was previously used and relied on feature level fusion for its input, in this work, CNNs were used to correspond with each of the five TISs where the inputs were the TIS data recorded by each corresponding TIS. A minor change to the approach was with regards to the number of pose classes. The differentiation between the left and right arm whenever the inhabitant was extending an arm outward was determined to be a redundant feature of the previous approach as, ultimately, it was only the extension of the arm itself that was required to understand the actions of the inhabitant. For this reason, the work presented in this paper does not include predictions for the specific arm that was being extended outwards during the completion of a pose, only that an arm was being extended outwards.

**Figure 1:**
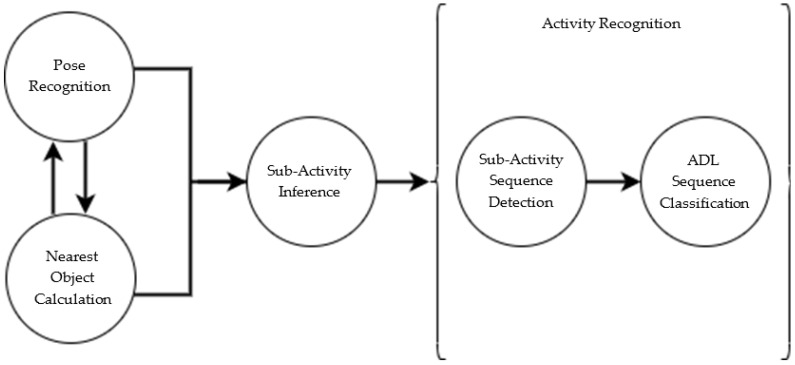
Our frame work for the production of an Activity of Daily Living (ADL) recognition approach where the pose recognition stage is investigated in this work.

This work is a low-level component of our overall proposed framework for ADL recognition, which is depicted in Figure 1. This paper expands upon the pose recognition approach to improve classification accuracy so that, ultimately, we will be able to improve upon our subactivity inference rate in future work when we investigate the use of subactivity sequences to detect and predict ADLs.

## 3. Materials and Methods

This section details the materials required for the experiment with regards to the sensors, the data and the CNN architecture. In addition, discussed are the methods employed to determine the most accurate permutation of TISs and CNNs that maintains a sufficient level of practicality.

### 3.1. Thermopile Infrared Sensor Details

**Figure 2:**
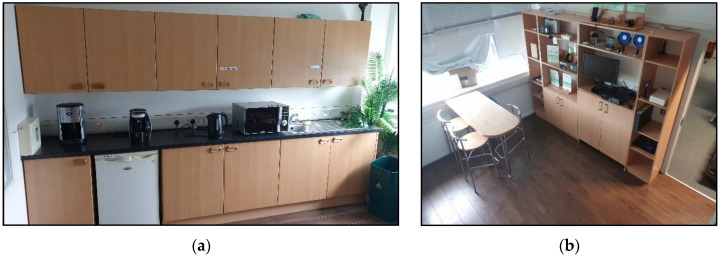
(**a**) Photograph capturing the kitchen bench, fridge and overhead cupboards within the smart environment and (**b**) photograph depicting the kitchen table and chairs in the smart environment.

A smart kitchen environment, located within Ulster University [26], was used for the deployment of TISs. Within the smart environment, there are common kitchen objects: kettle, microwave overhead cupboards, fridge and a kitchen table with four surrounding chairs. Images of the smart environment are presented in Figure 2.

**Figure 3:**
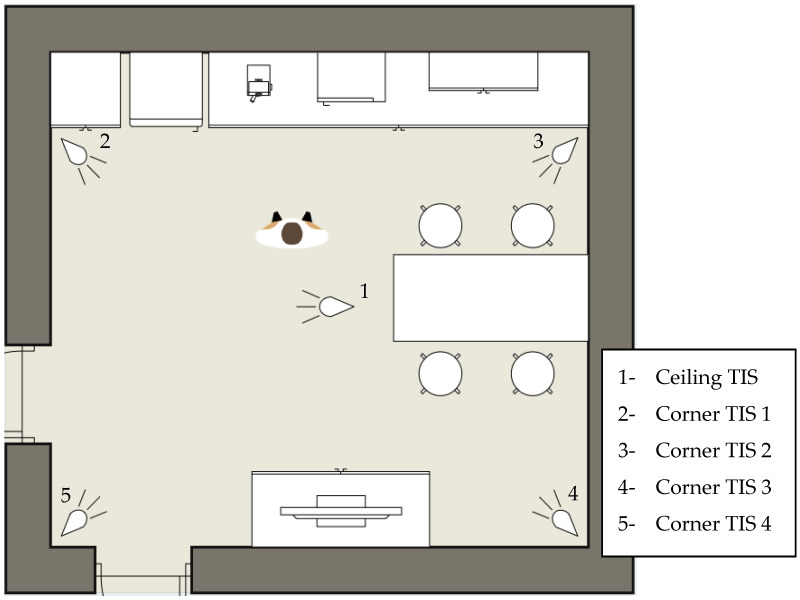
Top-down visualisation of the smart kitchen with labelled locations of each thermopile infrared sensors (TIS).

TISs were installed in each of the room’s four corners and one TIS was embedded in the ceiling. The deployment of TISs offered four lateral views of pose performances and one top-down view. A top-down perspective of the environment is visualised in Figure 3 where the positions of each TIS are labelled. The laterally positioned sensors were stabilised using tripods and were each maintained at the same height. The TISs were integrated with the SensorCentral [27] middleware, allowing for the captured thermal data to be exported as a JSON object.

Each TIS operated at a frame rate of approximately 8 frames per second. To provide synchronisation for the data capture, whenever images were recorded by the TISs, their timestamps were also stored for comparison. If there was a difference of 500 ms or more between the capture of any of the frames, the frame capture was not considered to have been synchronised and the frames were discarded.

### 3.2. Thermal Imagery for Training and Testing the CNN

The TISs were first used to capture the training data, which consisted of five pose classes: *Arm Forward*, *Arm Side*, *Arms Down*, *Bend* and *Sitting*. The classes were selected as the poses are commonly performed during the completion of activities within the kitchen. Accurate classification of the poses, therefore, could eventually be used to provide further knowledge with respect to the ADLs being performed. Examples of how each pose appeared to the TISs are presented in Table 1. The inhabitant performed the poses in the centre of the environment while periodically changing the direction they faced, allowing for the TISs to record variations in each pose class. The five TISs were used to record 500 unique instances of each pose class, resulting in a total of 2500 individual pose performances within the training dataset.

**Figure 4:**
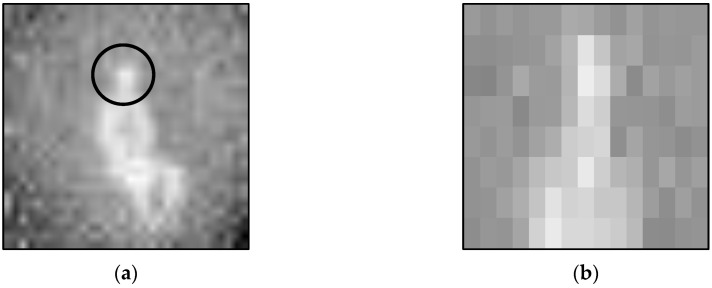
(**a**) TIS image depicting the sitting class where the circle indicates the region that is enlarged and (**b**) enlarged region of the inhabitant’s face.

The training data was captured over several days to account for varying ambient temperatures as a significant rise in the ambient temperature would increase the amount of image noise, consequently raising the difficulty of detecting a person. A significant decrease in the ambient temperature, however, would allow for easier detection of the inhabitant as there would be a greater difference between human body temperature and the temperature of the environment, along with the objects within it. There were, however, no considerable changes between the data captured on each day. Each training TIS image was manually reviewed and annotated with the pose class that was performed within the image. Manual annotation was also conducted for the test dataset in order to provide the ground truth with which the pose predictions for the test data could be compared for calculation of the experiment results. The capture of *Sitting* by C2 in Table 1 is depicted in Figure 4 where the image has been enlarged and zoomed in on the inhabitant’s face. The image exemplifies how the data used in this work was successful in protecting the privacy of the inhabitant as no definable characteristics of the inhabitant’s face are visible.

### 3.3. Architectural Design of the Convolutional Neural Network

In our previous work with TIS data, more traditional classifiers such as the random forest, SVM and decision tree were used with thermal imagery for classification purposes. The results that were achieved showed the approach to be successful, however, it was intended to improve upon the attained classification accuracy. The approach proposed within this work involved the deployment of additional TISs within the environment, to provide an increased number of viewing angles for each pose performance. It was also decided to investigate how CNNs could potentially improve the pose recognition rate achieved by the previously tested classifiers. This design choice was made due to the ability of CNNs to automatically extract low to high level features from raw image data for classification. In this work, five separate CNNs were trained, where each CNN corresponded with one of the five TISs. Each CNN was trained using only the thermal data captured by its respective TIS. Nevertheless, the CNNs maintained the same architectural design.

**Figure 5:**
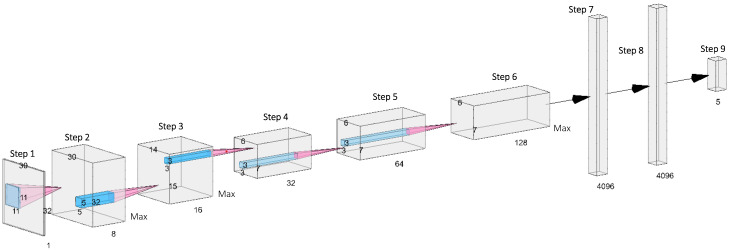
The proposed Convolutional Neural Network (CNN) architecture. Step one represents the image input layer, steps two to six represent the convolutional layers and their intervening pooled and normalisation layers and steps seven to nine show the fully connected layers. The cross-channel normalisation, batch normalisation, Rectified Linear Unit (ReLU) and dropout layers are not depicted here.

The CNN structure implemented for this experiment was initially based on the widely used AlexNet [28]. For our approach to pose recognition, modifications were made to the AlexNet architecture to improve the effectiveness of the network for the low-resolution thermal data captured by TISs. For a detailed explanation of AlexNet, readers may refer to [28]. Our CNN architecture is depicted in Figure 5, and it consisted of 30 layers in which eight were learnable: five convolutional and three fully connected. Unlike AlexNet, the output from the third fully connected layer was input into a five-way softmax layer as there were only five class labels rather than 1000. The only additional layer type included in our architecture, which is not used in AlexNet, was the batch normalisation layer. This addition facilitated faster network training due to the resulting higher learning rates, subsequently allowing for more efficient experimentation of the network. A batch normalisation layer was added after each of the five convolutional layers and before the activation functions, as such functions can result in non-Gaussian distributions [29].

Irrespective of the additional batch normalisation layers and the potential risk for overfitting that their inclusion created, the dropout layers used in AlexNet were maintained within our network. Experimentation also found that the omission of the dropout layers resulted in a worse training performance. A range of the hyperparameters for each convolutional layer were altered where the stride of the first convolutional layer was changed from four to one as irrespective of the detrimental impact that a smaller stride could have towards training speed, it ultimately favoured accuracy in comparison to a larger stride [30]. The number of filters for each of the five convolutional layers were reduced in AlexNet from 96, 256, 384, 384 and 256 to 8, 16, 32, 64 and 128, respectively. Due to the low-resolution images used in our approach, it was estimated that the number of patterns that could be detected from the data would be much lower than the RGB images used to train AlexNet. It was, therefore, hypothesised that it would be more appropriate to use a lower number of filters for the less complex low-resolution imagery. The number of filters increased with each new convolutional layer so that the deeper layers could effectively analyse the increasingly higher detail features, as performed with the architecture created in [30]. Like AlexNet, response-normalisation and max-pooling layers followed the first and second convolutional layers and a third max-pooling layer followed the fifth convolutional layer. The ReLU (Rectified Linear Unit) activation function was also maintained with AlexNet, however, the placement of the ReLU layers within our architecture was different. Instead of following each convolutional and fully connected layer, the ReLU layers followed each batch normalisation layer.

The image input layer was provided images of size 30 × 32 × 1 where the first convolutional layer applied 8 sliding convolutional filters of size 11 × 11 × 1 with a one-pixel stride. Due to the single pixel stride, the ‘same; padding hyperparameter used for the first convolutional layer in AlexNet was maintained so that the spatial output size remained the same as the input size for the layer. The second convolutional layer applied 16 sliding convolutional filters of size 5 × 5 × 8 to the output of the first convolutional layer once the output had passed through its respective pooling and normalisation layers. The third convolutional layer applied 32 sliding filters of size 3 × 3 × 16 to the pooled and response-normalised output of the second convolutional layer. The fourth convolutional layer used 64 sliding filters of size 3 × 3 × 32 where the fifth convolutional layer utilised 128 sliding filters of size 3 × 3 × 64.

### 3.4. Evaluation Methodology

The five TISs were used to capture a total of 2500 unique pose performances in which 500 frames of each of the five pose classes were recorded. As previously stated, each of the five TISs had a corresponding CNN where the CNNs were trained only with the frames captured by their respective TISs. The thermal data delegated to training each CNN was stratified and 10-fold cross-validation was implemented, leaving 90% of the data for training and 10% for validation. Each CNN was trained with its 90% training partition and validated with the 10% validation partition to give an indication of the capabilities to predict poses from the CNN’s respective TIS viewing angle. The process was repeated 10 times, allowing a different 10% partition of the data to act as the validation data each time, resulting in 10 models. The model that achieved the highest accuracy score with its respective validation partition was selected as the CNN for the particular TIS viewing angle to later apply to the test dataset for the final results. With respect to the checkpoint rule applied by the deep learning model throughout the training phase, the most recently updated model was saved, irrespective of the results it attained with the validation data. This training process was conducted for each of the TISs’ CNNs.

The test dataset was used to evaluate the performances of the trained CNNs. A total of 250 unique pose performances were captured for the test dataset where 50 frames of each pose class were included. Just as for the training dataset, the test dataset was captured from the five TIS viewing angles so that each CNN could be evaluated using only the data captured by its corresponding TIS. This test dataset was manually annotated to provide the ground truth to be used to produce the final results that are later presented in this paper.

### 3.5. Experimental Design

In our experiments, we investigated the use of CNNs to recognise poses performed in a smart kitchen. The assumption was made that the poses were conducted in the centre of the environment so that the pose classification capabilities of the CNNs could be evaluated with minimal occlusions of the pose performances. With a separate CNN trained for each TIS, a total of five trained CNNs were used for the experiment. Introducing five viewing angles into a pose recognition solution increased its robustness. If, e.g., a TIS’s view of the person was occluded or the TIS malfunctioned, the other TISs were in place to fulfil the classification.

As each CNN was trained only with data captured by its respective TIS, it was important to ensure that the pose instances were clearly depicted in each thermal frame. For instances of *Arm Forward* and *Arm Side*, it was possible that if the pose was performed with the arm extended towards the TIS, the pose may have looked more similar to the *Arms Down* class. The example frames in Table 1 show evidence of this. Upon capturing the training data, each frame was, therefore, manually checked to ensure that the pose was clearly depicted, however, if the pose was not, the image was replaced with one in which the pose class was clearly depicted. This was only conducted for the training dataset to improve the training of each CNN and no replacement frames were used for the test dataset. Using the trained CNNs in conjunction with one another, it was investigated how accurately the test data could be classified with regards to recall, specificity, precision and F1-score. An examination was conducted to find a permutation of the TISs that would be more in favour of cost, scalability and practicality, while still maintaining a high recognition rate.

For the experiments with the test dataset, the five frames that were captured during an instance of a pose performance were used to produce a prediction for the class of pose. Each TIS frame was input into its respective CNN and each CNN produced its own pose prediction. To produce the prediction output, the *softmax* layer converted a vector of real values, received from the previous layer, into a vector of values where each value represented one of the pose classes. The values summed to 1 so that they could be interpreted as probabilities. As each pose class was accompanied by a probability value, the class with the highest probability was selected as the CNN’s own pose prediction output. As each of the CNNs outputted a prediction for a single pose instance, a method for selecting one of the five predictions was required. A *Majority Vote* scheme was applied, where the class, which was predicted most among the five CNNs, was selected as the prediction for the pose instance.

Due to practical limitations, installation of the sensor configuration used in this experiment may not be possible in all environments. With respect to the ceiling TIS, the material or layout above the ceiling may not be fit for installing the TIS If an environment had a lower ceiling height than the environment in which the CNN’s were trained, the ceiling TIS’s FOV may not be sufficient for providing complete coverage of the environment. Detection of the inhabitant and their actions would, consequently, be limited. It would also be possible for the ceiling height to be higher than that of the training environment. If the ceiling was over 5 m, detection of the inhabitant’s heat signature would become more difficult for the ceiling TIS. Oversensorising the environment would also not be in the best interest of cost and postinstallation maintenance, especially if similar accuracies could be obtained with less TISs.

It is important to highlight that it is possible for an environment to be large enough that the corner TISs and CNNs could be subject to the same distance-based issues as the ceiling TIS. Unlike the ceiling TIS, however, the laterally positioned TISs are not limited to a fixed distance from the inhabitant and so the exact positioning of the TISs can be altered, dependent on the environment in which they are deployed. The task of installing and positioning lateral TISs was more practical than with a ceiling TIS and so the dependency on the ceiling TIS for pose recognition was omitted in further tests.

The pose classification capabilities of permutations of the corner TISs’ CNNs were tested, while excluding the ceiling TIS’s CNN from providing predictions due to the stated practical limitations. The ceiling TIS’s individual results were used as the benchmark as these results were the highest from any of the individual TIS performances. The *majority voting*, *Most Confident CNN* and *Soft Voting* prediction selection methods were implemented for the experiments and the results attained by each method were compared. The *Most Confident CNN* scheme was implemented by analysing the prediction probabilities generated by each of the five CNNs. As previously detailed, to provide a pose prediction, each CNN generated a probability distribution for the five pose classes, upon which the class with the highest corresponding probability value was selected as the CNN’s prediction. For the *Most Confident CNN* prediction selection approach, the CNN whose pose prediction was accompanied by the highest probability value was chosen as the most confident CNN and so its prediction was selected as the final prediction output for the pose instance.

The third method implemented was the *Soft Voting* approach. Given that each classifier represented a different viewing angle of the sensed environment and the inhabitant, weight values were assigned to each CNN as one may have been more capable of classifying the poses than the others. After each TIS image was input into the appropriate CNN, each CNN outputted a probability distribution where each probability corresponded with one of the five possible pose classes. The weight value assigned to a CNN was multiplied by each of the five pose probability values from the probability distribution, resulting in five weighted probabilities where each one corresponded with one of the five pose classes. After the weighted probabilities were calculated for each CNN probability distribution, the weighted probability values for each pose class were summed. For example, a weighted probability value was calculated for the *Arms Down* class by each CNN. Each of the *Arms Down* weighted probability values were summed, resulting in a single weighted sum value for the *Arms Down* class. A weighted sum value was calculated for each pose class and the class with the highest weighted sum was selected as the final prediction for the pose performance. The *Soft Voting* calculation for finding the weighted sum is shown in Equation (1) [31]:

(1) $$\hat{y}=\arg\underset{i}{\max}\overset{m}{\underset{j=1}{\sum }}w_{j}p_{ij},$$

where m is the number of CNNs, $w_{j}$ is the weight applied to the probability distribution that was generated by the j th CNN, $p_{ij}$ is the probability that class i is the correct class prediction according to CNN j and $\hat{y}$ is the class representing the largest weighted sum.

To determine the weight values for the classifiers, a naïve brute-force approach was employed [32]. In this approach, the range of values that could be used for the weights was determined by the number of CNNs involved. For example, if the CNNs for the four corner TISs were being used, then the values used for each CNN’s weight could only range from one to four. As each of the four CNNs had a possible four values to use as their weight, there was a total of 24 permutations of weight values that could be utilised. Each permutation of values for the weights were tested to find weights that yielded the most accurate pose predictions. This naïve approach to assigning weights was preferable over the use of the training results as upon capturing the training dataset and prior to training the CNNs, pose instances were replaced if they did not clearly depict their class. The validation data to produce the training results for each CNN, therefore, consisted only of favourable samples of each pose. The training results were not representative of how the CNNs would perform with the synchronised pose performances from the test dataset and so the weights could not be determined from the training results.

## 4. Results

In this section, the results obtained from implementing the deep learning architecture with five TIS viewing angles are presented. The individual results of each viewing angle and their respective CNNs are also detailed. A comparison is conducted, using the ceiling CNN as the benchmark, to find the most effective permutation of corner TISs. The approach to determining final pose predictions that subsequently provide the most accurate pose predictions is also investigated.

### 4.1. Training Results

For the training phase, each pose was captured with five TISs positioned at five different viewing angles where 500 frames for each pose class were recorded. A total of 2500 frames were, therefore, acquired for each class. If only one CNN was used to recognise poses, it would have been vital to ensure that the five TISs capturing a unique pose performance were doing so in a synchronised manner. This would have given an almost three-dimensional view of the performance. Nevertheless, it was decided to make use of a CNN for each TIS and train the networks with only frames captured from their respective TISs. It was not necessary, therefore, to enforce any manner of synchronisation to the training dataset, only that each frame was uniquely captured. The frames that did not clearly depict the pose that was being performed, such as instances of *Arm Forward* and *Arm Side* that looked more similar to *Arms Down*, were removed from the training dataset and appropriately replaced. This ensured that the features extracted from the frames were representative of the correct class and avoided confliction for the CNN during training.

As previously detailed, 10-fold cross-validation was utilised during the training of the CNNs where each CNN was trained individually. The results in Table 2 present the accuracies achieved by each CNN with their corresponding TIS’s perspectives of the pose performances within the training dataset. It is important to note, however, that very high training accuracies are achieved as each CNN is provided only with pose performances captured from their corresponding TISs, where the pose classes are clearly depicted within the frames. This is unlike a realistic scenario where a pose performance may be occluded and subsequently misclassified as there is no control over the orientation in which the inhabitant stands, therefore, multiple TIS viewing angles would be needed to produce an accurate pose prediction. The training results are not, therefore, truly representative of the recognition capabilities of the CNNs with unseen test data but, instead, an indication of successful training.

### 4.2. Experimental Results

The testing data was a separate dataset from the training dataset and was used to evaluate the performances of the trained CNNs. The data was recorded in such a manner to guarantee an equal number of frames for each class. There were 50 unique pose instances for each class captured from the 5 viewing angles for the testing data set. This provided 250 frames for each class. The testing data was captured on a different day and at a different time from the training data to allow for natural limitations on the dataset, such as the ambient temperature within the room. For the testing data, it was ensured that the thermal frames captured by the five TISs for a pose instance were synchronised, as they would be processed together towards generating a final prediction.

#### 4.2.1. Five TISs Test

To generate a prediction for each pose instance, the frames captured by each of the five TISs were input into their respective CNNs. Each of the five CNNs, therefore, produced a pose prediction for the pose instance, resulting in an interpretation, from five unique perspectives, of the pose being performed. This was done with the aim of increasing the likelihood of accurately recognising the poses. Only one of the five CNN outputs, however, could be selected as the final prediction and so a majority vote was held and of the five predictions, the class that was predicted the most was used as the final prediction for the pose instance. This prediction was then compared to the ground truth to determine its accuracy. The same was carried out for each of the remaining pose instances. Multiple final prediction selection methods were later compared, however, for the five TISs test, only the performance from the *Majority Vote* approach has been presented as it generated the highest accuracies by a significant margin.

Once all predictions were made and compared with the ground truth, scores for recall, precision, specificity and F1-score were calculated and can be viewed in Table 3, Table 4, Table 5 and Table 6, respectively. The result tables are structured in such a way as to show how well each TIS viewing angle performed, on their own, with each individual class and with all classes together. The results from when all TIS views were used together are presented in their own column.

Recall (Table 3) measures the probability that a prediction is a pose when the person is, in fact, performing that pose. This is the true positive rate. From the recall results, the ceiling TIS achieves the highest individual scores whenever all poses were included, however, C2 performed better than the ceiling CNN for *Sitting*. There is a potential advantage for some classifiers to view *Sitting* from an adjacent perspective as the ceiling CNN could classify the pose incorrectly as *Bend*. The performance by C2 did not, however, surpass the other TISs in every other pose as C1 recognised *Arms Down* the best and C3 recognised *Arm Side* the best. The highest scorer among the corner TISs for *Arm Forward* was C4, however, this TIS and CNN combination also resulted in the lowest recall score for *Arms Down* which was significantly lower than the other results. This demonstrates the need for multiple TISs as where one may fail to produce an accurate prediction, another may correctly recognise the pose instance, therefore, providing a level of robustness to the approach.

Precision (Table 4) is similar to the recall in that it measures the probability that the person is performing a pose whenever the classifier is predicting the same pose. This is important as there may be cases where, e.g., the recall for a pose is 1.000, however, several other frames were also predicted as the same pose irrespective of belonging to a different class. The precision score indicates how many times a pose class was predicted when the pose was actually being performed. The results again show the ceiling CNN to have given the best results for when all poses were judged together. C1 outperformed Ceiling on *Arm Side* and this, along with the recall results, reveal that while the ceiling CNN accurately predicted *Arm Side* whenever the inhabitant was performing the pose, there were several cases where other poses were incorrectly classified as *Arm Side*. This alludes to the fact that none of the classifiers were faultless when operating individually, as is shown by the 0.1250 increase to 1.000 for *Arm Side* when all TISs were then used together.

The lowest precision score for all poses was made by C4, where the CNN attained precision results for the *Sitting* class that were significantly lower than the other CNNs. These low scores were caused by the misclassifications of *Arms Down* as *Sitting* by the C4 CNN where only 28% of *Arms Down* instances were correctly classified. The C4 CNN also frequently misclassified *Arms Down* as *Arm* Forward, subsequently contributing to the low precision score for *Arm Forward*, however, misclassifications of *Arm Side* as *Arm Forward* were the primary cause of this. From the perspective of C4, the classes are alike, resulting in both classes being mistaken for the other. This also repeatedly occurred for C2 and C3, further signifying how difficult it is to provide accurate predictions for all classes with a single corner TIS. As there is no guarantee as to which TIS will experience classification difficulties, this furthers the necessity for multiple TISs.

Specificity (Table 5) measures the probability that a classifier will not predict a pose whenever the pose is not being performed. This is the true negative rate. For each class, the specificity considers the number of times the class was rightfully not predicted, and the correct class was instead predicted. The lowest specificity achieved when all poses were judged together was by C4. The poor classification of *Arms Down* continued to negatively impact the recognition scores of the C4 CNN as instances of *Arms Down* were often misclassified as *Arm Forward* and *Sitting*, causing the poses to be predicted whenever they were not actually being performed. This subsequently caused the low specificity results. The C4 CNN did, however, perform similarly to the other CNNs with respect to its predictive capabilities for four of the classes, therefore, indicating that while a model can underperform with respect to some of the classes, maintaining its inclusion for providing prediction outputs can still prove to be complementary for predicting other classes.

The F1-score (Table 6) is a function of the recall and the precision and expresses the balance between the two measurements. These test results show how differently each TIS, on its own, can perform. These large differences could have been caused by varying temperatures throughout the room that were a product of sunlight and appliances. Such cases created more areas of high temperature within the image, resulting in more image noise. This may have caused some images to be misclassified as this issue would have been possible in both the training and testing data. The results demonstrate how certain TISs perform well on classes where others do not and vice versa, therefore, the need for various viewing angles of the environment is exemplified further. This is the case for C1 and C2 as C1 achieves a much higher F1-score than C2 in the Arm Side class, whereas for Sitting, C2 obtains a higher F1-score than C1. Due to this complementary nature, it is, therefore, desirable to use all five TISs. The confusion matrix generated from the test can be found in Table 7.

The values in the cells represent the number of frames (out of 250) that were predicted to be the class, which is in the header of the respective column. The cells that are positioned diagonally through the matrix where the predicted class label and true class label match, therefore, indicate the number of correctly predicted frames for the respective class. The other cells provide insight for frames that were incorrectly predicted with regards to what the incorrect prediction and the actual class label were.

#### 4.2.2. Thermopile Infrared Sensor Permutation Tests

While the results achieved by the deep learning approach are promising, the argument could be made that deploying many TISs may not be the most practical solution and so a reduction in the sensor count was investigated. From the results, it was clear that the ceiling TIS was, by a large margin, the best performing TIS with regards to single individual performances. The results it achieved were very close to the results from the five TISs test. It is, however, important to address the possible limitations of relying on the predictive capabilities of the ceiling TIS.

The ceiling height within the environment used to capture the training data is not representative of all possible ceiling heights, and clearly the height of different environments’ ceilings will vary. Issues could arise from ceilings higher than 10 m as a person’s heat signature would start to become difficult for the TIS to detect. Ceilings too low could create difficulties for the TIS to capture, within a single frame, the whole pose performed by an inhabitant, causing the poses to appear vastly different from the poses the CNNs were trained with. This would lead to an undesirable scenario where an undertrained CNN would be playing an important role in classification tasks. It was, therefore, decided to exclude the ceiling TIS and corresponding CNN from holding a responsibility to generate predictions and instead, aimed to only use the corner TISs.

While the width and length of a room may differ just as the height of a ceiling may differ, the use of laterally positioned TISs instead of ceiling-based TISs would make the deployment of numerous TISs more practical and any necessary reorientation more possible. This is due to the existing practical challenges with the installation of ceiling TISs. Such challenges may not be able to be overcome in some environments, therefore, limiting both the number of TISs that could be integrated within the environment as well as the number of positions on the ceiling capable of housing a TIS. Numerous TISs would ultimately benefit the prediction capabilities as frames of a pose could be captured from multiple unique perspectives and subsequently, the frames could be analysed in conjunction with one another to better predict the class. Due to the underperforming individual performances of the corner TISs, various permutations of the TISs were investigated to find the best performing and most cost-effective permutation. The ceiling view’s individual results were used as the benchmark for these tests as it provided the highest individual results and was the only TIS omitted from the remaining experiments.

##### Four Thermopile Infrared Sensors Test

The predictions made by the CNNs corresponding with the four corner TISs were passed through *Majority Vote*, *Most Confident CNN* and *Soft Voting* prediction selection approaches; Table 8 presents the results.

The results show that both the *Majority Vote* and *Most Confident CNN* approaches performed similarly, while the *Soft Voting* approach achieved the highest scores with an F1-score of 0.9266. The naïve method of finding the combination of weight values that yielded the highest scores allowed for the approach to be tested more effectively than the two lower scoring methods. The confusion matrix produced from this approach is presented in Table 9.

##### Three Thermopile Infrared Sensors Test

Investigating the use of only three corner TISs was the next test carried out. This involved not only finding the best approach to prediction selection but also the best permutation of three corner TISs. Each possible permutation of three TISs was tested and it was found that a different permutation scored highest for each prediction selection approach. The highest scoring results for each approach along with the respective permutations are presented in Table 10. Only the highest scoring permutations from each approach are included as the comparison is conducted to determine the best prediction selection technique for three corner TIS permutations.

The *Soft Voting* approach again generated the highest recognition rate of the poses. It is also promising that there is just a difference of 0.0117 between the F1-score achieved by the four corner TISs and the F1-score achieved by the three corner TISs. This was identified to be a result of the low individual performance of C4 and how it did not contribute many correct predictions that were not already made by C1, C2 and C3. Its omission for the utilisation of the three corner TISs, therefore, did not cause a significant decrease in correct predictions. This approach, however, did not achieve as promising results for the recognition of *Arm Side* as it did for the other poses, as can be seen in the confusion matrix in Table 11. The *Arm Side* F1-scores attained through the use of the *Majority Vote* and *Most Confident* CNN approaches were 0.4600 and 0.7200, respectively. The *Soft Voting* approach, however, attained an F1-score of 0.8095 for *Arm Side*, therefore, demonstrating its better performance even for its lowest scoring class.

##### Two Thermopile Infrared Sensors Test

Finally, two corner TISs were used to try to achieve results close to the F1-score benchmark of 0.9478. Again, the prediction selection approach and corner TIS permutation that performed best were investigated. As there were only two predictions available to select a final prediction from, the *Majority Vote* approach was not tested. It was found that for both approaches, the same TIS permutation of C1 and C2 was the best; Table 12 presents the scores this permutation achieved.

The similarity between the recognition rates achieved by both approaches is evident and, therefore, requires a deeper analysis to determine the best prediction selection scheme. The confusion matrices generated from each approach can be found in Table 13 and Table 14. The shortcomings from both approaches were the same with low recognition rates of *Arm Forward* and *Arm Side*. With its marginal improvement over the recall and F1-score of the *Most Confident CNN* approach, the *Soft Voting* approach was selected as the best approach for the two corner TIS test.

As is the case, the highest scoring permutation was expected to have consisted of two adjacent TISs rather than TISs positioned directly opposite one another. This was due to adjacent TISs complementing one another in providing a more realistic coverage of the monitored space and the poses being performed within it. Using two opposite TISs negated the potential benefits of using multiple TISs as, e.g., whenever *Arm Forward* was performed, if the person was facing one of the TISs, neither TIS would have been able to view the outstretched arm. The same applied to the *Arm Side* class.

##### Permutation Comparison

The highest F1–scores achieved by the four, three and two TIS permutations were 0.9266, 0.9149 and 0.8468, respectively. While the four corner TIS method produced the score closest to the benchmark, the three corner TIS method produced a similarly high score with one less TIS. The comparisons between the TIS permutations and the benchmark are presented in Table 15. The use of a ceiling TIS is not necessarily a priority due to the three alternatives proving to be more than suitable. Having three alternative options, with each differing in their number of TISs, is highly desirable when aiming to use thermal imagery for recognition-based tasks.

**Figure 6:**
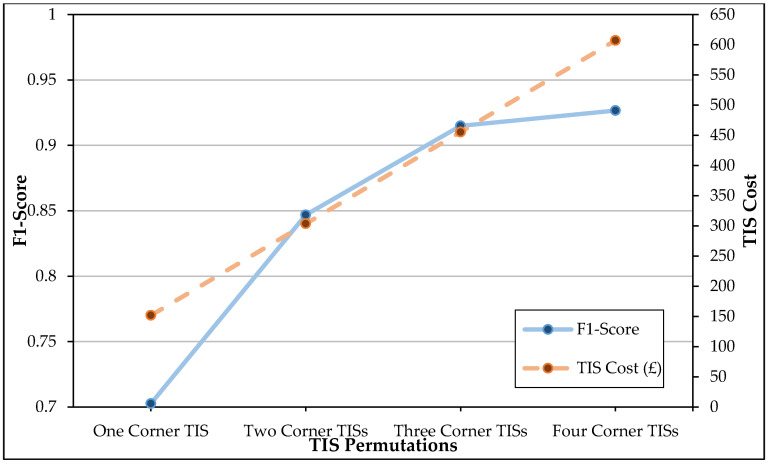
Trade-off between the number of TISs and CNNs, performance and cost of the TISs.

The trade-off between the number of TISs, the achieved F1-score and the TIS cost is visualised in the chart in Figure 6. A very high efficiency can be achieved using only four and three corner TISs as an almost identical F1-score to the benchmark was achieved, falling short only by 0.0212 and 0.0329, respectively. If a task required a more simplistic deployment with only two TISs, the F1-score would be sacrificed by 0.1010, however, the practical and financial benefits of only deploying and purchasing two TISs could, in the appropriate context, outweigh the loss in accuracy. The best individual performance from using only one corner TIS is achieved using C1 and its corresponding CNN. The use of a single corner TIS requires the same cost as the ceiling TIS while avoiding the practical limitations involved with installation. This is, therefore, the least expensive and most practical implementation. Nevertheless, the F1-score is 0.2452 lower than the benchmark and 0.1442 lower than the two TIS permutation. Due to the significantly lower recognition rate, an increase in cost can be justified to obtain results considerably closer to the benchmark.

##### Permutation Results with a Random Forest Model

The random forest machine learning algorithm used in our previous work was incorporated for the same tests conducted with the CNNs in this study. In our previous work, we tested several machine learning algorithms, however, the random forest was the highest scoring, therefore, its classification capabilities of the dataset presented in this study were chosen to compare with the CNN. While the datasets used in this study differ from our previous work, the approach to training and testing the random forests with TIS data remained the same. Nevertheless, the random forest required training for pose recognition and, like the CNNs, a random forest model corresponded with each TIS. Manual selection and extraction of features for each pose performance was necessary and for further details on our training and testing approach with the random forest, please refer to [25].

The same tests involving the same datasets were conducted where the only difference was the use of random forest models instead of CNNs. The results achieved with the best scoring permutations of random forest models and respective TISs are presented in Table 16.

Permutations of four, three and two TISs with corresponding random forest models achieved F1-scores of 0.7980, 0.8523 and 0.8379, respectively. None of the random forest models, however, achieved a score higher than the benchmark with the closest score being achieved by the three TIS permutation, attaining an F1-score that was 0.1237 less than the ceiling benchmark. The CNNs performed better in this respect as two permutations of CNNs achieved F1-scores over 0.9000 where the highest was only 0.0212 less than the benchmark. The benchmark achieved with the ceiling random forest model was higher than with the ceiling CNN, however, as already established, a ceiling-based TIS was not as practical as laterally based TISs and so this improvement was negligible. For permutations of three and four TISs, the CNNs improved upon the random forests by a considerable margin. The 2 corner TIS permutation was the only permutation in which similar scores were achieved with both machine learning methods, however, the recognition of the individual classes indicated that the CNN produced a better performance. The results presented in Table 17 compare the individual pose class F1-scores achieved by both the machine learning models with the two corner TIS test. While the random forests achieved a significantly higher F1-score for *Arms Down*, the CNNs achieved a more significant improvement for the F1-score of *Arm Side.*

**Figure 7:**
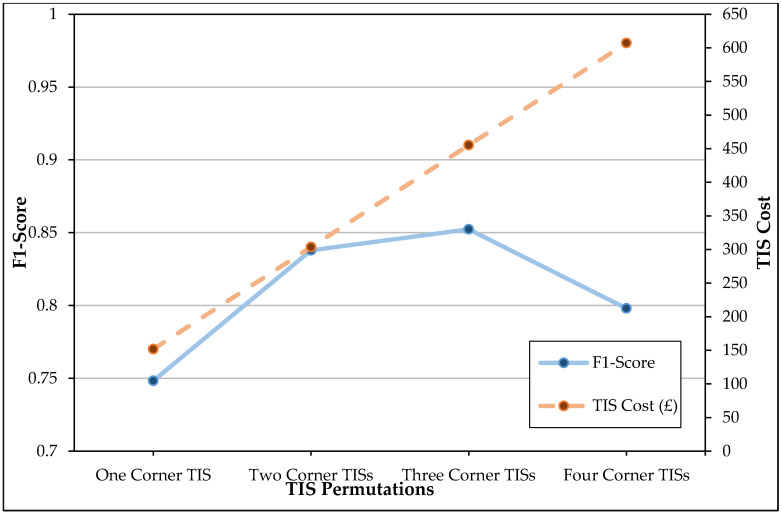
Trade-off between the number of TISs and random forests, performance and cost of the TISs.

A trade-off between the number of TISs, pose recognition performance and TIS cost is visualised in Figure 7 to investigate whether a more cost-effective and practical solution justifies the significant performance loss experienced with the random forest models. Unlike with the use of CNNs, a steady increase in the permutation cost did not produce a similarly steady increase in pose recognition performance with the random forest models. Whenever the cost was at its highest with four corner TISs, the performance of the random forest models resulted in one of the lowest F1-scores, second only to the F1-score attained by a single corner TIS. While the three corner TIS permutation performed better than both the four and two corner TIS permutations, it only improved upon the F1-score of the two corner TIS permutation by 0.0144 while costing £151.78 more. The F1-score attained with three random forests was also 0.0626 less than what was achieved with three CNNs. The two corner TIS permutation, however, performed considerably better than the single corner TIS, therefore, justifying its cost. The two corner TIS permutation was, therefore, the most cost-effective permutation of corner TISs when corresponding random forest models were implemented.

## 5. Discussion

The aim of this study was to investigate how effectively unobtrusive thermal imagery could be used to train a deep learning network to recognise poses in previously unseen data, while preserving the privacy of the inhabitant. The deep learning network was inspired by AlexNet where several modifications were made to benefit the low-resolution imagery captured by the TISs and improve the classification results. The Long Short-Term Memory (LSTM) architecture was considered for this work, however, as the problem involved the classification of single poses, the advantage of capturing temporal dependencies between different input images that the LSTM would provide was less relevant. A TIS was installed in the ceiling and each of the room’s four corners. The five TISs were used to capture an equal number of frames for each of the five pose classes. Each TIS’s captured data was used to train a CNN, resulting in five CNNs trained to recognise poses from the viewing angle of their respective TIS. Upon finishing the training phase, unseen thermal data were used to test the CNNs. Much like in the training dataset, the test dataset was made up of five viewing angles for each pose performance. The TIS frame from each viewing angle was, therefore, input into its respective CNN. The output from this process was five pose predictions which were narrowed down to one final prediction using a *Majority Vote* scheme. This use of five TISs returned a high F1-score of 0.9920, demonstrating the successful implementation of the deep learning network for pose recognition.

The use of five TISs allowed for multiple perspectives of each pose performance and added robustness to the approach. The lateral perspectives provided by the corner TISs aided the ceiling TIS whenever it struggled to either detect the person or differentiate between poses with similar appearances. For example, from the perspective of the ceiling TIS, bending and sitting looked very similar and so the lateral perspectives helped differentiate between the classes. The ceiling TIS provided an important contribution with its strong capability to recognise whenever the person was extending an arm outwards and whether this was sideward or forward.

Beyond the successful pose classification that was achieved with five TISs, the most practical and cost-effective permutation of TISs was determined. The ceiling TIS did not fit the model of practicality due to the difficulty involved in deploying the TIS in an unobtrusive manner, e.g., ensuring no cables were visible. The F1-score from the ceiling TIS’s individual performance was the highest among all five TISs and so it was used as the benchmark with which to compare the performances of corner TIS permutations. There can be concern for environments varying in width and length, potentially causing the same distance-related issues suffered by the ceiling TIS for ceilings of varying height. For the laterally positioned TISs, however, the sensors are not limited to a fixed distance from the inhabitant. If necessary, it would be possible to position the TISs closer to where it is expected that the inhabitant will be most active.

From using four, three and two corner TISs, the CNNs achieved F1-scores of 0.9266, 0.9149 and 0.8468, respectively. These are promising results as the permutations of four and three corner TISs reached F1-scores that were very similar to that of the benchmark, suggesting that both accuracy and practicality can be achieved. A financial drawback, however, is that more than one corner TIS was required to achieve these scores. While the two-TIS permutation’s recognition rate was not as close to the benchmark, it uses only one additional TIS and so is a more cost-effective sensor deployment. The 0.0681 decrease from the three-TIS permutation and 0.1010 decrease from the benchmark could be considered as a justifiable sacrifice to ensure a more practical and financially sound approach. Selecting between the permutations will, however, ultimately depend on the context and resources at hand.

The experiments were conducted a second time where the CNN architecture was replaced with a random forest machine learning algorithm used in our previous work. The results were compared with the CNN results, and it was found that the best F1-score for a single corner TIS was achieved with a random forest model. The score was, however, still too low to justify the use of only one corner-based TIS, irrespective of the low cost. The CNNs significantly outperformed the random forests with three and four corner TIS permutations, however, a similar result was achieved with the permutation of two corner TISs. With respect to the individual pose class scores, however, the F1-scores attained by CNNs were more favourable. As a more promising balance between performance, practicality and cost can be achieved with the use of CNNs and several permutations of corner TISs, our previous classification approach has been successfully improved upon.

In each experiment, TISs were used as the only means for monitoring the environment and capturing pose performances. The sole use of TISs allowed for the preservation of privacy in our approach. While multiple thermal images were recorded of the inhabitant during each pose performance, the low-resolution and greyscale nature of the images prevented any discernible characteristics of the inhabitant from being visible. As an improvement upon other privacy-preserving approaches, image processing techniques are not required to hide an inhabitant’s face and there is no manner of accessing identifiable features of the inhabitant should the system be hacked. There is, however, a significant loss in environmental detail through the utilisation of low-resolution images. Nevertheless, only changes in the shape of the inhabitant’s heat signature is required for our approach and this is sufficiently accomplished with the use of the TISs. Privacy has, therefore, been successfully preserved with our approach to collecting data within a smart environment for pose recognition.

## 6. Conclusions

High classification scores have been produced while preserving privacy with the various approaches proposed in this work, however, limitations are present with respect to the data capture process and the sizes of both the training and test datasets. The thermal data captured for the experiments sufficiently accounts for pose classes that are commonly performed throughout the completion of ADLs, as well as the variable appearances of each pose that are dependent on where the inhabitant is facing relative to the TISs. The sizes of the datasets are, however, limited as the training data consist of only 500 instances of each class, while the test dataset consists only of 250 total pose performances. A concern is that a larger dataset may expose further limitations in the work through negatively impacting the recognition results. In further work, we will aim to increase the size of the datasets by capturing more data or by employing data augmentation. The datasets will also be expanded with respect to the number of environments in which poses are performed. Evaluating the proposed approach with the inclusion of other environments will facilitate the testing of additional poses and activities, as well as different positions of the laterally based TISs relative to the inhabitant.

The process of capturing the pose instances for the training and test datasets was limited due to the assumption that the inhabitant would only stand in the centre of the room to perform the selected poses while rotating in varying directions. This assumption was made so that the TISs would have an unobstructed view of the pose performances. The poses could have instead been performed under realistic conditions such as interacting with the environment during the completion of ADLs. Nevertheless, the aim for this work was to improve upon our previous classification approach, while also establishing an understanding of the capabilities for predicting poses, using multiple viewing angles in conjunction with the proposed CNN architecture. Further work is required to determine the predictive capabilities of CNNs when movement of the inhabitant, subtle pose performances, and occlusions of the inhabitant are each considered. In future work, pose instances for both training and testing will, therefore, involve interactions between the inhabitant and the environment, such as *Arm Forward* at the microwave or *Bend* at the fridge.

The results achieved with the thermal imagery can be improved still with the integration of image segmentation techniques. In future work, active contour-based segmentation techniques will be investigated for application to the TIS data to allow for simpler detection of the inhabitant. In future work, we will also investigate the inclusion of abnormal behaviour within the dataset to test whether such behaviour could be differentiated from poses and activities. Successful implementation of abnormal behaviour detection will help indicate whether TISs are suitable devices for deployment within care homes as such behaviour can aid in the diagnosis of age-related diseases.

As deep learning has been successfully implemented with the TISs to recognise poses, this will be expanded to establish a deep learning-based methodology for recognising ADLs. As shown by our previous work, the ability to recognise poses has proven to be extremely useful for inferring subactivities such as using the fridge and sitting at the kitchen table. It is intended to implement an HMM to work in conjunction with the CNNs detailed in this research to classify sequences of subactivities as ADLs. Both a deep learning and a more conventional machine learning approach will be implemented for performance comparisons. This work will continue to use TISs as the only sensory data sources so that privacy can continue to be preserved.

## Figures and Tables

**Table 1:** Examples of the five classes as seen from each thermopile infrared sensor (TIS) viewing angle.

Pose	Ceiling Sensor	C1 Sensor	C2 Sensor	C3 Sensor	C4 Sensor
Arms Down	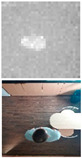	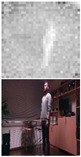	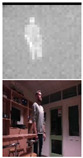	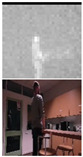	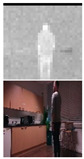
Bend	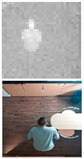	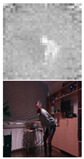	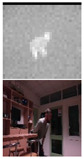	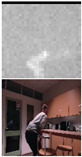	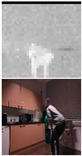
Arm Forward	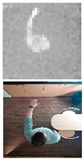	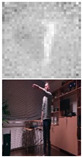	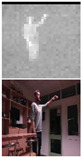	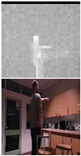	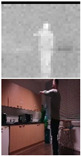
Arm Side	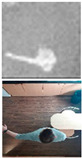	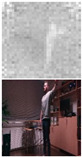	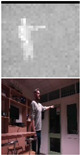	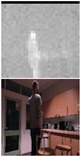	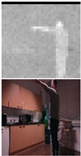
Sitting	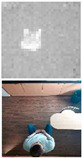	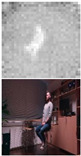	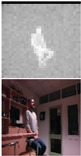	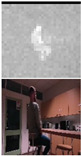	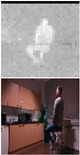

**Table 2:** The average accuracy over 10 folds for each Convolutional Neural Network (CNN) along with the average for all five CNNs together where C = corner CNN.

Classifier	Maximum Accuracy Achieved over 10 Folds (%)
Ceiling	99.80
C1	99.92
C2	99.92
C3	99.92
C4	99.96
All Views	99.90

**Table 3:** Recall results from the five TISs test where C = corner.

Class	Ceiling	C1	C2	C3	C4	All Views
Arm Forward	0.8400	0.4800	0.5000	0.4800	0.5200	1.0000
Arm Side	0.9800	0.6000	0.3600	0.6200	0.3200	0.9800
Arms Down	0.9800	0.9000	0.7000	0.6200	0.2800	0.9800
Bend	0.9800	0.9800	1.0000	1.0000	1.0000	1.0000
Sitting	0.9600	0.5600	0.9800	0.7200	0.7800	1.0000
All Poses	0.9480	0.7040	0.7080	0.6880	0.5800	0.9920

**Table 4:** Precision results from the five TISSs test where C = corner.

Class	Ceiling	C1	C2	C3	C4	All Views
Arm Forward	1.0000	0.9231	0.6944	1.0000	0.4262	1.0000
Arm Side	0.8750	1.0000	0.6923	0.4306	0.5333	1.0000
Arms Down	0.9250	0.5056	0.5469	0.6200	0.8750	0.9800
Bend	0.9800	0.6533	0.7463	0.7813	0.6849	1.0000
Sitting	0.9800	0.9333	0.8596	0.9000	0.5571	0.9804
All Poses	0.9518	0.8031	0.7079	0.7464	0.6153	0.9921

**Table 5:** Specificity results from the five TISs test where C = corner.

Class	Ceiling	C1	C2	C3	C4	All Views
Arm Forward	1.0000	0.9870	0.9325	1.0000	0.7727	1.0000
Arm Side	0.9641	1.0000	0.9521	0.7747	0.9021	1.0000
Arms Down	0.9792	0.7486	0.8304	0.8813	0.9850	0.9950
Bend	0.9947	0.8301	0.8819	0.8971	0.8051	1.0000
Sitting	0.9947	0.9867	0.9412	0.9714	0.7737	0.9950
All Poses	0.9865	0.9105	0.9076	0.9049	0.8477	0.9980

**Table 6:** The F1-score results from the five TISs test where C = corner.

Class	Ceiling	C1	C2	C3	C4	All Views
Arm Forward	0.9130	0.6316	0.5814	0.6486	0.4685	1.0000
Arm Side	0.9245	0.7500	0.4737	0.5082	0.4000	0.9899
Arms Down	0.9515	0.6475	0.6140	0.6200	0.4242	0.9800
Bend	0.9800	0.7840	0.8547	0.8772	0.8130	1.0000
Sitting	0.9697	0.7000	0.9159	0.8000	0.6500	0.9901
All Poses	0.9478	0.7026	0.6879	0.6908	0.5511	0.9920

**Table 7:** Confusion matrix generated from testing all five TISs together.

True Class	Predicted Class				
	Arm Forward	Arm Side	Arms Down	Bend	Sitting
Arm Forward	50	–	–	–	–
Arm Side	–	49	1	–	–
Arms Down	–	–	49	–	1
Bend	–	–	–	50	–
Sitting	–	–	–	–	50

**Table 8:** Pose recognition results achieved using the four corner TISs with three methods of prediction selection.

Selection Method	Recall	Precision	Specificity	F1-Score
Majority Vote	0.8560	0.8699	0.9595	0.8524
Most Confident CNN	0.8480	0.8658	0.9590	0.8492
Soft Voting	0.9280	0.9354	0.9813	0.9266

**Table 9:** Confusion matrix generated from implementing the *Soft Voting* approach to prediction selection for four corner TISs.

True Class	Predicted Class				
	Arm Forward	Arm Side	Arms Down	Bend	Sitting
Arm Forward	48	–	2	–	–
Arm Side	4	37	9	–	-
Arms Down	–	–	47	–	3
Bend	–	–	–	50	–
Sitting	–	–	–	–	50

**Table 10:** Pose recognition results achieved using permutations of three corner TISs with three methods of prediction selection.

Selection Method	TIS Permutation	Recall	Precision	Specificity	F1-Score
Majority Vote	C1, C2, C3	0.8520	0.8626	0.9593	0.8484
Most Confident CNN	C1, C2, C4	0.8480	0.8686	0.9588	0.8488
Soft Voting	C1, C2, C3	0.9160	0.9308	0.9786	0.9149

**Table 11:** Confusion matrix generated from implementing the *Soft Voting* approach to prediction selection for three corner TISs.

True Class	Predicted Class				
	Arm Forward	Arm Side	Arms Down	Bend	Sitting
Arm Forward	49	–	1	–	–
Arm Side	–	34	16	–	–
Arms Down	–	–	46	1	3
Bend	–	–	–	50	–
Sitting	–	-	–	–	50

**Table 12:** Pose recognition results achieved using permutations of two corner TISs with three methods of prediction selection.

Selection Method	TIS Permutation	Recall	Precision	Specificity	F1-Score
Most Confident CNN	C1, C2	0.8400	0.8910	0.9578	0.8430
Soft Voting	C1, C2	0.8440	0.8953	0.9590	0.8468

**Table 13:** Confusion matrix generated from implementing the *Most Confident CNN* approach to prediction selection for two corner TISs.

True Class	Predicted Class				
	Arm Forward	Arm Side	Arms Down	Bend	Sitting
Arm Forward	32	–	18	–	–
Arm Side	1	34	15	–	–
Arms Down	–	–	48	2	–
Bend	–	–	–	50	–
Sitting	–	–	–	4	46

**Table 14:** Confusion matrix generated from implementing the *Soft Voting* approach to prediction selection for two corner TISs.

True Class	Predicted Class				
	Arm Forward	Arm Side	Arms Down	Bend	Sitting
Arm Forward	32	–	18	–	–
Arm Side	1	34	15	–	–
Arms Down	–	–	49	1	–
Bend	–	–	–	50	–
Sitting	–	–	–	4	46

**Table 15:** The comparison of achieved recognition scores between the best scoring approaches for each TIS permutation and the benchmark.

TIS Positions and Quantities	TIS Permutation	Recall	Precision	Specificity	F1-Score
Ceiling (Benchmark)	Ceiling	0.9480	0.9518	0.9865	0.9478
One Corner TIS	C1	0.7040	0.8031	0.9105	0.7026
Two Corner TISs	C1, C2	0.8440	0.8953	0.9590	0.8468
Three Corner TISs	C1, C2, C3	0.9160	0.9308	0.9786	0.9149
Four Corner TISs	C1, C2, C3, C4	0.9280	0.9354	0.9813	0.9266

**Table 16:** The comparison of attained recognition scores between the best scoring approaches for each TIS permutation and the benchmark when using the random forest model.

TIS Positions and Quantities	TIS Permutation	Recall	Precision	Specificity	F1-Score
Ceiling (Benchmark)	Ceiling	0.9760	0.9763	0.9939	0.9760
One Corner TIS	C3	0.7560	0.7980	0.9282	0.7483
Two Corner TISs	C1, C2	0.8480	0.8888	0.9594	0.8379
Three Corner TISs	C1, C2, C3	0.8600	0.8868	0.9619	0.8523
Four Corner TISs	C1, C2, C3, C4	0.8240	0.8824	0.9539	0.7980

**Table 17:** Comparison between the F1-scores attained by the random forest and CNN models during the two corner TIS test.

Class	Random Forest	CNN
Arm Forward	0.7460	0.7711
Arm Side	0.5915	0.8095
Arms Down	0.9320	0.7425
Bend	0.9600	0.9524
Sitting	0.9600	0.9583
All Poses	0.8379	0.8468

## References

[1] Cook D.J., Das S.K. (2007). How smart are our environments? An updated look at the state of the art. Pervasive Mob. Comput..

[2] United Nations, Department of Economic and Social Affairs, Population Division (2019). World Population Prospects.

[3] Wiles J.L., Leibing A., Guberman N., Reeve J., Allen R.E.S. (2012). The Meaning of “Aging in Place” to Older People. Gerontologist.

[4] Abowd G., Bobick A., Essa I., Mynatt E., Rogers W. (2002). The Aware Home: A living laboratory for technologies for successful aging. Automation as Caregiver: The Role of Intelligent Technology in Elder Care, Proceedings of the AAAI-02 Workshop on Edmonton, Alberta, Canada, 29 July 2002.

[5] Rantz M., Skubic M., Miller S., Galambos C., Alexander G., Keller J., Popescu M. (2013). Sensor Technology to support Aging in Place. J. Am. Med. Dir. Assoc..

[6] Arifoglu D., Charif H., Bouchachia A. (2020). Detecting indicators of cognitive impairment via Graph Convolutional Networks. Eng. Appl. Artif. Intell..

[7] Wallace M., Shelkey M., Hartford Institute for Geriatric Nursing (2008). Katz Index of Independence in Activities of Daily Living (ADL) Katz Index of Independence in Activities of Daily Living. Urol. Nurs..

[8] Zubair M., Song K., Yoon C. Human activity recognition using wearable accelerometer sensors. Proceedings of the IEEE International Conference on Consumer Electronics-Asia.

[9] Kartsch V., Benatti S., Mancini M., Magno M., Benini L. Smart Wearable Wristband for EMG based Gesture Recognition Powered by Solar Energy Harvester. Proceedings of the IEEE International Symposium on Circuits and Systems.

[10] Crandall A., Cook D. (2011). Tracking systems for multiple smart home residents. Human Behaviour Recognition Technologies.

[11] Demiris G., Rantz M.J., Aud M.A., Marek K.D., Tyrer H.W., Skubic M., Hussam A.A. (2004). Older adults’ attitudes towards and perceptions of ’smart home’ technologies: A pilot study. Inform. Health. Soc. Care.

[12] Chen L., Hoey J., Nugent C.D., Cook D.J., Yu Z. (2012). Sensor-based activity recognition. IEEE Trans. Syst. Man Cybern. Part C Appl. Rev..

[13] Liciotti D., Frontoni E., Zingaretti P., Bellotto N., Duckett T. HMM-based Activity Recognition with a Ceiling RGB-D Camera. Proceedings of the International Conference on Pattern Recognition Applications and Methods.

[14] Gaglio S., Re G.L., Morana M. (2015). Human Activity Recognition Process Using 3-D Posture Data. IEEE Trans. Hum.-Mach. Syst..

[15] Schapire R.E. (2003). The Boosting Approach to Machine Learning: An Overview. Nonlinear Estimation and Classification.

[16] Goodfellow I., Bengio Y., Courville A. (2016). Deep Learning.

[17] Su H., Maji S., Kalogerakis E., Learned-Miller E. Multi-view Convolutional Neural Networks for 3D Shape Recognition. Proceedings of the IEEE International Conference on Computer Vision.

[18] Deng J., Dong W., Socher R., Li L.-J., Kai L., Li F.-F. ImageNet: A large-scale hierarchical image database. Proceedings of the IEEE Conference on Computer Vision and Pattern Recognition.

[19] Wu Z., Song S., Khosla A., Yu F., Zhang L., Tang X., Xiao J. 3D ShapeNets: A Deep Representation for Volumetric Shapes. Proceedings of the IEEE Computer Society Conference on Computer Vision and Pattern Recognition.

[20] Pittaluga F., Zivkovic A., Koppal S.J. Sensor-level privacy for thermal cameras. Proceedings of the IEEE International Conference on Computational Photography.

[21] Griffiths E., Assana S., Whitehouse K. (2018). Privacy-preserving Image Processing with Binocular Thermal Cameras. Proc. ACM Interact. Mob. Wearable Ubiquitous Technol..

[22] Capgo—Sensor Glossary. http://www.capgo.com/Resources/Sensors/SensorGlossary.html.

[23] Heimann Welcome to Heimann Sensor’s Website. http://www.heimannsensor.com/thermopile-arrays.

[24] Quero J., Burns M., Razzaq M., Nugent C., Espinilla M. (2018). Detection of Falls from Non-Invasive Thermal Vision Sensors Using Convolutional Neural Networks. Proceedings.

[25] Burns M., Morrow P., Nugent C., McClean S. (2019). Fusing thermopile infrared sensor data for single component activity recognition within a smart environment. J. Sens. Actuator Netw..

[26] Nugent C.D., Mulvenna M.D., Hong X., Devlin S. (2009). Experiences in the development of a Smart Lab. Int. J. Biomed. Eng. Technol..

[27] Rafferty J., Synnott J., Ennis A., Nugent C., McChesney I., Cleland I. (2017). SensorCentral: A research oriented, device agnostic, sensor data platform. Ubiquitous Computing and Ambient Intelligence.

[28] Krizhevsky A., Sutskever I., Hinton G.E. (2017). ImageNet Classification with Deep Convolutional Neural Networks. Commun. ACM.

[29] Ioffe S., Szegedy C. Batch normalization: Accelerating deep network training by reducing internal covariate shift. Proceedings of the International Conference on Machine Learning.

[30] Sermanet P., Eigen D., Zhang X., Mathieu M., Fergus R., LeCun Y. Overfeat: Integrated recognition, localization and detection using convolutional networks. Proceedings of the International Conference on Learning Representations.

[31] EnsembleVoteClassifier—Mlxtend. http://rasbt.github.io/mlxtend/user_guide/classifier/EnsembleVoteClassifier/.

[32] Raschka S. Implementing a Weighted Majority Rule Ensemble Classifier. https://sebastianraschka.com/Articles/2014_ensemble_classifier.html.

